# Smartphone-assisted spatial data collection improves geographic information quality: pilot study using a birth records dataset

**DOI:** 10.4081/gh.2016.482

**Published:** 2016-11-23

**Authors:** Xiaohui Xu, Hui Hu, Sandie Ha, Daikwon Han

**Affiliations:** 1 Departament of Epidemiology and Biostatistics, Texas A&M University, College Station, TX; 2 Departament of Epidemiology, University of Florida, Gainesville, FL, USA

**Keywords:** Positional accuracy, Geocode, Vital statistics, Bias, Environmental epidemiology

## Abstract

It is well known that the conventional, automated geocoding method based on self-reported residential addresses has many issues. We developed a smartphone-assisted aerial image-based method, which uses the Google Maps application programming interface as a spatial data collection tool during the birth registration process. In this pilot study, we have tested whether the smartphone-assisted method provides more accurate geographic information than the automated geocoding method in the scenario when both methods can get the address geocodes. We randomly selected 100 well-geocoded addresses among women who gave birth in Alachua county, Florida in 2012. We compared geocodes generated from three geocoding methods: i) the smartphone-assisted aerial image-based method; ii) the conventional, automated geocoding method; and iii) the global positioning system (GPS). We used the GPS data as the reference method. The automated geocoding method yielded positional errors larger than 100 m among 29.3% of addresses, while all addresses geocoded by the smartphone-assisted method had errors less than 100 m. The positional errors of the automated geocoding method were greater for apartment/condominiums compared with other dwellings and also for rural addresses compared with urban ones. We conclude that the smartphone-assisted method is a promising method for perspective spatial data collection by improving positional accuracy.

## Introduction

Geocoded, vital statistics birth records have been widely used to examine the potential adverse effects of environmental exposures during pregnancy on pregnancy and birth outcomes, including low birth weight, preterm delivery, small for gestational age (Dadvand *et al*., 2012; Metcalfe *et al*., 2011; Sapkota *et al*., 2012; Shah and Balkhair, 2011; Stieb *et al*., 2012; Strand *et al*., 2011), congenital anomalies (Vrijheid *et al*., 2011), pregnancy complications such as hypertensive disorders of pregnancy (Hu *et al*., 2014), and gestational diabetes mellitus (Hu *et al*., 2015). A wide range of environmental factors have been investigated in previous studies, including air pollution (Hu *et al*., 2014, 2015; Sapkota *et al*., 2012; Shah and Balkhair, 2011; Stieb *et al*., 2012; Vrijheid *et al*., 2011), temperature (Strand *et al*., 2011), greenness (Dadvand *et al*., 2012), built environment (Hystad *et al*., 2014; Miranda *et al*., 2012), and other neighbourhood-level factors such as income, education, and racial residential segregation (Anthopolos *et al*., 2014; Metcalfe *et al*., 2011). These studies provide important evidence in this field. However, geocoded information in the vital statistic birth records using the traditional automated, geocoding method based on self-reported residential addresses has many issues including missing geocode data and positional errors of geocoded addresses.

**Figure 1:**
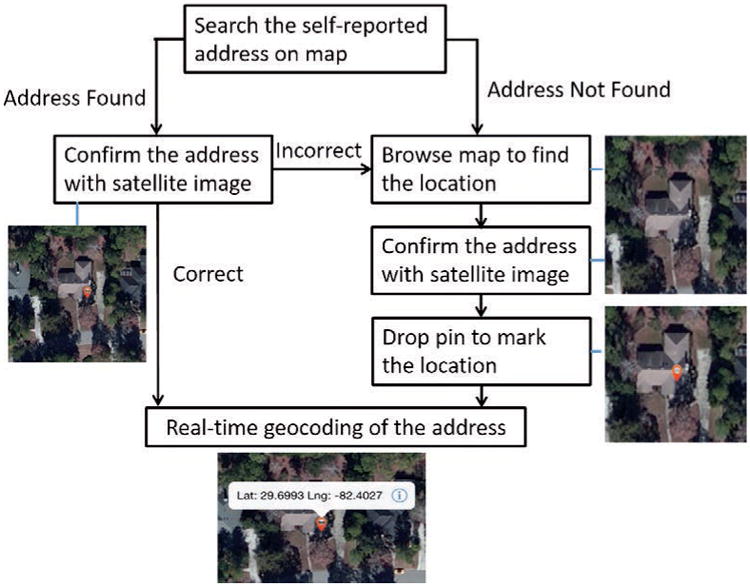
Illustration of a smartphone-assisted aerial image-based method for spatial data collection

The issues regarding positional accuracy of geocoded addresses have drawn much attention and recent studies suggest that potential errors cannot be ignored when using geocoding methods in epidemiologic studies (Cayo and Talbot, 2003; Hurley *et al*., 2003; Whitsel *et al*., 2006). The positional errors seen with geocoding can have substantial impacts on many salient factors underlying environmental epidemiologic studies (Jacquez, 2012), including exposure estimates (Zandbergen, 2007), health access analysis (Frizzelle *et al*., 2009; McLafferty *et al*., 2012), disease cluster detection (Jacquez and Rommel, 2009; Zimmerman *et al*., 2008), disease rates estimates (Goldberg and Cockburn, 2012), and spatial weights (Jacquez and Rommel, 2009). More importantly, studies have shown the heterogeneity in positional accuracy with greater geocoding errors observed in rural compared to urban areas (Cayo and Talbot, 2003; Hurley *et al*., 2003; Whitsel *et al*., 2006). These errors may cause a differential mis-classification among rural and non-rural individuals and lead to biased results in epidemiologic studies (Krieger *et al*., 2001; Oliver *et al*., 2005). Alternative geocoding methods such as aerial image-based methods have been available for a long time and are usually used for improving positional accuracy of addresses in the traditional *post-hoc* geocoding method. The advantages of these methods have been reported by many authors (Baltsavias, 1993; Boulos, 2005; Conzelmann *et al*., 2005; Hild and Fritsch, 1998; Richards *et al*., 1999; Ward *et al*., 2005), but limited knowledge regarding the addresses among geographic information system technicians could significantly restrict their application in geocoding. More importantly, to our knowledge, these techniques have not been used for spatial data collection. We propose a smartphone-assisted aerial image-based method for spatial data collection during the process of birth registry. This method has many advantages including map/aerial image searching for addresses, participants' involved verification and real-time geocoding over the traditional *post-hoc* geocoding method (Figure 1). The prospective use of such methods has the potentials to substantially improve data quality by reducing missing values and improving the accuracy of geographic information.

In this pilot study, we aimed to examine if the smartphone-assisted, aerial image-based method provides more accurate geographic information than the *post-hoc* geocoding method in the scenario when both methods can obtain the geographic information of an address.

## Materials and Methods

### Study population and geocoding by Florida Department of Health

We obtained birth record data from the Bureau of Vital Statistics & Office of Health Statistics and Assessment, Florida Department of Health (FDOH), Tallahassee, FL, USA. The data included all registered live births in Florida (FL), USA between January 1, 2012 and December 31, 2012 (n=211,437). The FDOH used ArcGIS 10.1 software with the topologically integrated geographic encoding and referencing (TIGER) street database from the US Census Bureau to geocode maternal residential address at delivery for all FL residents, while 1,093 births with maternal address outside FL were not geocoded. A total of 206,796 (98.3%) women were successfully geocoded among the 210,344 women living within the state of Florida. A total of 2733 women with geocoded maternal residential addresses inside Alachua county, FL were eligible to be sampled in this study. The population of Alachua county was 251,417 (71% urban, 29% rural) that year. From these eligible addresses, a total of 100 addresses were statistically randomly sampled using the SURVEYSELECT procedure in SAS 9.3 (http://support.sas.com/documentation/cdl/en/statug/63962/HTML/default/viewer.htm#statug_surveyselect_sect001.htm).

We compared geocodes generated from three geocoding methods: i) the conventional, FDOH-geocoded records using an automated, geocoding method based on the TIGER street database (https://www.census.gov/geo/maps-data/data/tiger.html) and ArcGIS (http://www.esri.com); ii) reference measures using global positioning system (GPS) receivers 5 m away from the sampled addresses (outside the building); and iii) the geocodes obtained from the smartphone-assisted, aerial-based method using the Google Maps application programming interface (API) (Google, 2015).

### Global positioning system receiver measurements

The Garmin GPSMAP^®^ 76Cx receiver (Garmin International Inc., Olathe, KS, USA) was used. The typical position accuracy of this receiver ranges from 3 to 5 m, and it has been validated and widely used in many studies (Wing, 2008). In this study, GPS measurements were taken 5 m away from the sampled addresses (outside the building), in order to avoid direct interactions or contacts with any residents. None of the addresses located in apartment complexes have controlled access during daytime when the measurements were done. All data were collected in January 2015.

### The smartphone-assisted, aerial image-based method

Besides the automated and GPS-measured geocodes, we developed and used a method built on satellite and aerial images using Google Map API (Google, 2015). Briefly, the researchers automatically search the address on the map, browse the aerial images, verify the location (*i.e*. simulating the process of participant-involved verification) and obtain the geocodes of the address, or the first placed pinpoint on the aerial images if the address cannot be automatically found, aligned with the centroid location of each actual address. The system then returns and records the longitude and latitude for the pinpoint. Figure 1 shows the algorithm of the smartphone-assisted, aerial image-based method for spatial data collection during participant interview. As shown, the geographic coordinates of the location will be automatically generated and collected from this proposed method so that no *post-hoc* data cleaning or geocoding is needed. In this pilot study, the data collectors all had background knowledge obtained through field visits to the selected addresses that served as participants.

### Covariates

Information of maternal, socio-demographic status was obtained from the vital statistics dataset, including maternal age at delivery (<30 or ≥30 years old), race (black or non-black), education level (<high school, high school, or >high school), marital status (married or not married) and insurance types (Medicaid or non-Medicaid). In addition, housing types were categorized into two groups: apartment/condominium and others. We also categorised each address as urban or rural based on the GPS-measured geocodes using the 2013 cartographic boundary shapefiles (urban areas) from the US Census (https://www.census.gov/geo/maps-data/data/cbf/cbf_ua.html).

### Statistical analysis

The geocodes measured by the GPS receiver were used as the reference in this study. Geocodes from all three different methods were based on the datum WGS84. The positional errors of the automated geocoded addresses by FDOH and the geocodes generated using the smartphone-assisted method were determined by their geodetic distance (the shortest path along the ellipsoid of the Earth at sea level between two points) to the GPS-measured geocodes in meters using the GEODIST function in SAS 9.3. Descriptive statistics were generated where appropriate, and paired t-tests were used to examine the difference in positional errors between the automated geocoding method and the smartphone-assisted method. The distribution of parcel size for the addresses was generated by housing type (apartment/condominium or not). We used both regression and tree-based methods to model the potential association between housing types, maternal characteristics, urbanization and the positional accuracy of the automated geocoding method. The positional errors of the automated geocoded addresses by FDOH were modelled both as continuous and dichotomous variables (>100 m or 100 m). The cut-off of 100 m was selected because of its widely use in literatures of positional accuracy and environmental exposure assessment (Bonner *et al*., 2003; Gordian *et al*., 2006; Wu *et al*., 2005; Zandbergen *et al*., 2011). We first fitted generalized linear models for these outcomes and all covariates with the continuous outcomes log-transformed to account for its skewed distribution, and then used regression trees to further explore the potential interactions and nonlinear association between the covariates and the outcomes (James *et al*., 2013). The regression tree is a non-parametric method which recursively partitions the data space and fits a simple prediction model within each partition. Therefore, it can identify complex interaction and non-linear associations between the predictors and the outcome without any *a priori* specification. Data management was performed using SAS 9.3 and all analysis were conducted using R 3.1.2.

## Results

Among the 100 randomly sampled addresses, 99 were successfully identified and geocoded using both the GPS receiver and the smart-phone-assisted method. All subsequent analyses were based on the 99 successfully identified and geocoded addresses. For the one remaining address, apparent errors in the street number made it unidentifiable, so it was excluded from this study.

Table 1 shows the distribution of maternal socioeconomic status at delivery, housing and area characteristics. Most of the women living in the sampled addresses were less than 30 years old (65.66%), Non-Black (64.65%), had education levels greater than high school (74.75%), married (59.60%) or had insurance other than Medicaid (61.62%). Approximately 30% of the housing was apartments or condominiums and approximately 14% of the addresses were located in rural areas. Table 1 also presents the geometric means of positional errors measured by both the automated geocoding method and the smartphone-assisted method. Overall, the automated geocoding method yielded a mean (geometric) positional error of 56.46 m, while the error for the smartphone-assisted method was confined to 13.30 m. Consistent patterns were observed in all subgroups by scociodemographic status, housing and area characteristics. In addition, the paired t-test showed significant differences between all pairs examined (all P values <0.05). The distribution of parcel size by housing type is presented in Table 2.

**Figure 2:**
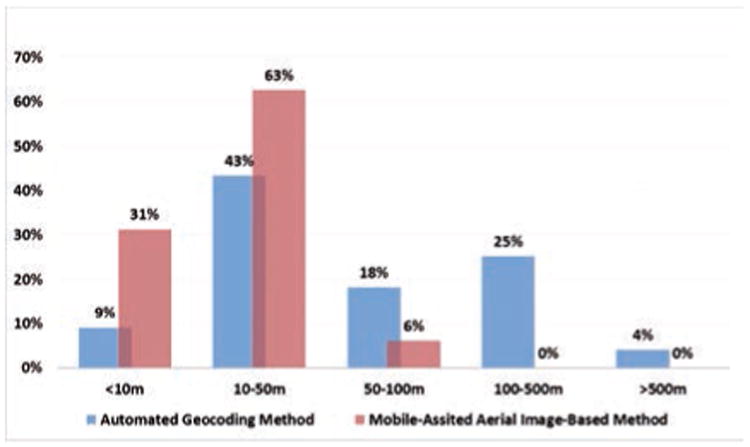
The positional errors between the automated geocoding method and the smartphone-assisted method

Figure 2 compares the positional errors between the automated geocoding method and the smartphone-assisted method. All aerial image geocoded locations fell within 100 m away from the true location with around 94% of them within 50 m. However, only around 70% of the automated geocoded addresses were within 100 m of the true location with 52 and 9% having errors less than 50 and 10 m, respectively. When stratified (Table 3), we found higher proportions of mis-classified addresses for apartment/condominiums compared with other housing types (67 *vs* 13% of addresses with positional errors greater than 100 m) and when comparing addresses located in rural areas to those located in urban areas, the outcome was 43 *vs* 27%, respectively, when the automated geocoding method was used for geocoding. In addition, there was no address with >100 m positional errors with the new mobile-assisted method.

Table 4 shows the results of the generalized linear models used to examine the potential association between the positional errors of the automated geocoding method and covariates. The continuous model showed that the housing type of apartment/condominium was associated with a 1.59 [95% confidence interval (CI): 1.07, 2.12] increase in the log-transformed positional error. In addition, the logistic regression model found that addresses of the apartment/condominium housing type compared with those located in rural areas had 64.54 (95% CI: 14.94, 409.55) and 9.66 (95% CI: 1.79, 64.93), respectively, times the odds of being automatically geocoded with positional errors >100 m, respectively. Nonblack women's addresses were also found to be significantly associated with an increased odds ratio (OR: 7.08, 95% CI: 1.25, 51.90) of having positional errors greater than 100 m when using the automated geocoding method.

**Figure 3:**
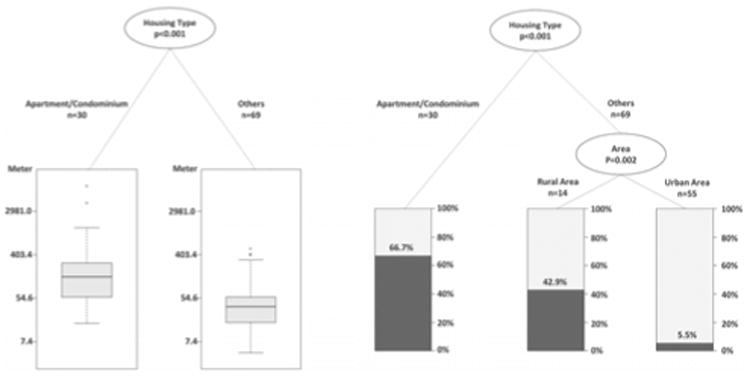
Covariates significantly associated with positional errors of the automated geocoding method

Figure 3 presents the covariates significantly associated with positional errors of the automated geocoding method from the regression trees analyses. The housing type was significant in both models on continuous and dichotomous outcomes and urbanity was shown as an important predictor for positional errors of the automated geocoding method among the addresses that were not apartment/condominiums.

## Discussion

Using GPS receivers as the reference measure for true location, we compared the positional errors of the automated geocoding method used by FDOH and the smartphone-assisted geocoding method. The conventional automated geocoding method has substantial deficiencies in positional accuracy with approximately 30% of the geocoded addresses having positional errors exceeding 100 m; this is a significant methodologic shortcoming in many settings of environmental epidemiologic studies (Griffith *et al*., 2007; Zandbergen, 2008). The positional errors of the automated geocoding method observed in this study are comparable to previous research conducted in the states of Iowa, New York and Texas, from where 21-28% of the automated geocoded addresses over 100 m have been reported (Bonner *et al*., 2003; Ward *et al*., 2005; Zhan *et al*., 2006). More importantly, our study shows that such errors are not randomly distributed given the association observed between positional errors and housing type and urbanity. In addition to the urban-rural heterogeneity of positional errors reported from previous studies (Cayo and Talbot, 2003; Hurley *et al*., 2003; Whitsel *et al*., 2006), we observed even larger heterogeneity among addresses referring to apartment/condominiums. These non-randomly distributed errors may lead to a differential misclassification bias that will greatly influence the validity of studies based on these automated geocoding data.

In addition, we found that the smartphone-assisted geocoding method may substantially increase the positional accuracy compared with traditional geocoding. Different from some previous studies which used the geocodes by the aerial image as the true location *gold standard* (Schootman *et al*., 2007), we regarded aerial image as a potential method for address location verification during the spatial data collection. Although the aerial image substantially improved positional accuracy, it still had slightly discrepancy when compared with the GPS-measured geocodes. This may be due to several reasons, of which the resolution of the aerial image is one important factor. In addition, in our study, some of the homes could not be accurately identified in the aerial images since they were covered and surrounded by trees and green spaces. In spite of these limitations, the smartphone-assisted method still offered significant improvement over the traditional methods, especially for addresses for apartment/condominiums since most automated geocoding methods cannot handle apartment-level information.

Extensive efforts have been devoted to improve automated geocoding, and many methods have been proposed including the manual intervention (Chaput *et al*., 2002; Goldberg *et al*., 2008; Ward *et al*., 2005), re-geocoding with a different geocoder (Lovasi *et al*., 2007; Zhan *et al*., 2006), and imputation or pseudocoding (Boscoe, 2008; Henry and Boscoe, 2008; Strickland *et al*., 2007). However, all these methods focused on improving spatial data quality after the data collections. The proposed smartphone-assisted method integrates the aerial image-based manual corrections to the data collections, thus making it possible to prospectively collect and geocode addresses, to verify the geocoded data during data collections, which is particularly important.

Previous studies have suggested an error rate of 10% and a missing rate of 5% of self-reported addresses in public health surveillance datasets (Zinszer *et al*., 2010). Such errors and missing data can be caused by both participants and administrative staff. Participants may accidentally skip or report a wrong address due to many reasons such as privacy concerns and recall errors. On the other hand, staff may make data-entry and processing mistakes. Importantly, the automated geocoding method may sometimes fail to identify such errors and even assign a *false-matched* geocode. Unfortunately, it is hard to detect such errors in large datasets and there is no existing validation tool to identify and fix these errors in the data collection process. Such errors are therefore almost impossible to correct once the data collection has been completed. However, this proposed smartphone-assisted method can avoid these issues during the process of data collection with participants' involved verification, real-time of geocoding and aerial image/map-assisted real time search. This proposed method can easily be integrated into many data collection systems and so obtain high-quality spatial data. Integrations of this method into data collection systems will transfer the efforts of geocoding from the data collectors to the participants, making it feasible for data collection in large health studies or electronic health records such as vital statistics birth records. It will also allow participants to interact with this geocoding system directly offering an unprecedented use of street maps, satellite images and street views to reduce missing records as well as to improve positional accuracy. Indeed, participants have more local knowledge than GIS technicians and can accurately verify and find the locations of their addresses on maps/aerial images. Therefore, the use of this method for spatial data collection has a great potential with respect to improving spatial data quality.

Several limitations of this study should be noted. First, this is a pilot study that has a relatively small sample size and focused on only one county. Additionally, the smartphone-assisted method was conducted by researchers. Ideally, residents may provide more accurate geocoding information using the system, as they are more familiar with the neighbourhood, especially when the home cannot be directly identified in the image. Furthermore, measurement errors may exist for the reference method using GPS receiver since we were not able to enter the participants' homes.

## Conclusions

With respect to the vital statistics birth record dataset, studies relying on automated geocoding may suffer from potential differential bias. Addresses referring to the housing apartment or condominium type and addresses located in rural areas are more likely to have greater positional errors. The smartphone-assisted method may substantially improve positional accuracy in geocoding, which has the potential to be used as a spatial data collection tool to further improve spatial data quality.

## Figures and Tables

**Table 1:** Geometric means of positional errors by maternal scociodemographic status and housing and area characteristics.

Parameter	N	%	Positional error (m), geometric mean±SD		P
			Automated geocoding method	Mobile-assisted aerial image-based method	
Total	99	100.00	56.46±3.81	13.30±3.18	<0.001

Age at delivery (years)					
<30	65	65.66	58.22±3.98	11.92±3.18	<0.001
≥30	34	34.34	53.25±3.56	16.41±3.14	<0.001

Race					
Black	35	35.35	45.57±3.44	10.15±3.60	<0.001
Non-black	64	64.65	63.48±4.05	15.43±2.90	<0.001

Education					
<High school	17	17.17	55.33±4.24	10.83±3.43	<0.001
High school	8	8.08	59.88±4.39	9.47±4.58	0.031
>High school	74	74.75	56.36±3.74	14.47±3.01	<0.001

Marital status					
Married	59	59.60	67.20±3.83	13.76±3.36	<0.001
Not married	40	40.40	43.67±3.68	12.67±2.96	<0.001

Insurance					
Medicaid	38	38.38	48.90±3.61	10.72±3.51	<0.001
Non-Medicaid	61	61.62	61.75±3.95	15.22±2.95	<0.001

Housing type					
Apartment/condominium	30	30.30	151.09±4.06	7.91±3.28	<0.001
Other	69	69.70	36.80±2.90	16.68±2.93	<0.001

Area					
Urban area	85	85.86	54.97±3.85	12.94±3.14	<0.001
Rural area	14	14.14	66.40±3.69	15.72±3.56	0.021

SD, standard deviation.

**Table 2:** Distribution of parcel size (square meters) by housing type.

Housing type	N	Median	Mean	SD	Quartile 1	Quartile 3
Apartment/condominium	30	40,984.13	57,131.70	62,680.05	1627.07	104,800.82
Others	69	1104.41	18,958.34	48,402.67	730.78	7265.93
Total	99	1390.65	24,742.18	52,294.61	801.27	20,234.57

SD, standard deviation.

**Table 3:** Positional errors by housing type and area.

Housing type/area	Total number of addresses	Automated geocoding method		Mobile-assisted aerial image-based method	
		Addresses with errors >100 m	%(95% CI)	Addresses with errors >100 m	% (95% CI*)
Apartment or condominium	30	20	66.67 (49.80, 83.54)	0	-

Other	69	9	13.04 (5.10, 20.99)	0	-

Urban area	85	23	27.06 (17.61, 36.50)	0	-

Rural area	14	6	42.86 (16.93, 68.78)	0	-

CI, confidence interval.

**Table 4:** Associations between positional error of automated geocoding method by Florida Department of Health and maternal socioeconomic status and housing characteristics.

Parameter	Continuous (Log-transformed), β(95% CI)	Dichotomous (>100 m *vs* ≤100 m), OR (95% CI)
Age at delivery (years)		
<30	Reference	Reference
≥30	-0.25 (-0.79, 0.30)	0.89 (0.22, 3.70)

Race		
Black	Reference	Reference
Non-black	0.32 (-0.36, 1.00)	7.08 (1.25, 51.90)

Education		
<High school	Reference	Reference
High school	0.37 (-0.54, 1.28)	4.92 (0.50, 53.51)
>High school	0.38 (-0.40, 1.15)	0.63 (0.08, 5.09)

Marital status		
Married	Reference	Reference
Not married	-0.43 (-1.09, 0.23)	0.77 (0.14, 4.23)

Insurance		
Medicaid	Reference	Reference
Non-Medicaid	0.02 (-0.66, 0.69)	0.42 (0.08, 2.09)

Housing type		
Apartment/condominium	Reference	Reference
Other	1.59 (1.07, 2.12)	64.54 (14.94, 409.55)

Area		
Urban area	Reference	Reference
Rural area	0.62 (-0.12, 1.35)	9.66 (1.79, 64.93)

CI, confidence interval; OR, odds ratio.
